# Beyond Dysphagia in Parkinson’s Disease: 3D Printing of Orally Disintegrating Tablets (ODTs) for Optimized Treatment

**DOI:** 10.3390/ph18121886

**Published:** 2025-12-13

**Authors:** Ying-Ju Liao, Yao-Jen Liang

**Affiliations:** 1 Department of Research and Development, Merdury Biopharmaceutical Corporation, New Taipei City 235030, Taiwan; 2 Institute of Traditional Medicine, School of Medicine, National Yang Ming Chiao Tung University, Taipei 112304, Taiwan; 3 Department of Life Science, Fu-Jen Catholic University, New Taipei City 242062, Taiwan; 4 Graduate Institute of Applied Science and Engineering, Fu-Jen Catholic University, New Taipei City 242062, Taiwan

**Keywords:** Parkinson’s disease, 3D printing, dysphagia, orally disintegrating tablets

## Abstract

Parkinson’s disease (PD) is frequently complicated by dysphagia, undermining timely and reliable oral dopaminergic medication. Levodopa’s short half-life and delayed gastric emptying in advanced PD result in inconsistent absorption, delayed “ON” periods, and challenges to adherence. Orally disintegrating tablets (ODTs) dissolve without water and can mitigate swallowing limitations. Research indicates that selegiline ODT achieves a faster time to peak and higher relative bioavailability via partial buccal absorption, whereas carbidopa/levodopa ODTs are bioequivalent to immediate release tablets (with similar AUC/C_max_ and approximately 1 h T_max_) without consistent motor advantages but with higher patient acceptability. This review synthesizes the clinical burden of dysphagia in PD, pharmacokinetic constraints of current formulations, and the reasons for ODTs. We highlight 3D printing as a route to personalized, dysphagia friendly therapy, which enables dose individualization, polypills, engineered disintegration or release, and point-of-care production. Feasibility studies underscore stability considerations such as carbidopa, throughput and regulatory requirements (QbD/GMP), and bioequivalence information. We outline priorities to integrate 3D printed ODTs into PD care, aligning formulation, pharmacokinetics, and human factors to improve adherence and clinical outcomes.

## 1. Introduction

Parkinson’s disease (PD) is a prevalent neurodegenerative movement disorder that manifests with both motor and non-motor symptoms. Approximately 0.93 million people were living with PD in the United States in 2020, with projections of about 1.24 million by 2030 [1]. As more than half of the world’s elderly population resides in Asia, PD posing a significant public health challenge in countries such as Taiwan, China, and Japan [2]. As PD progresses, patients commonly develop dysphagia (swallowing difficulty), a non-motor symptom with severe health implications [3]. Dysphagia occurs in more than one-third of PD patients on average (pooled prevalence: 37%) and up to 70–87% in advanced cases depending on the assessment method [3,4]. This impairment leads to the malnutrition, dehydration, and aspiration of food or pills into the airway, often causing aspiration pneumonia. Aspiration pneumonia is the leading cause of death in PD, responsible for roughly 25% of PD-related mortality [5]. Difficulties in swallowing interfere with quality of life and limit medicine administration [4]. Managing motor symptoms typically requires multiple daily doses of immediate-release (IR) carbidopa/levodopa (approximately 5 times daily), which not only increases treatment complexity but also affects patient medication adherence [6]. Levodopa is a dopamine precursor that crosses the blood–brain barrier to alleviate motor symptoms, whereas carbidopa is a peripheral decarboxylase inhibitor given alongside levodopa to prevent its conversion to dopamine outside the brain. This combination increases the amount of levodopa reaching the central nervous system (CNS) and reduces peripheral side effects [7].

Oral levodopa remains the gold-standard therapy for PD motor control, but effective use of levodopa is challenged by late-stage disease progression and gastrointestinal dysfunction. In advanced PD, loss of physiologic dopamine buffering and delayed gastric emptying led to erratic absorption and motor response fluctuations [8,9]. Patients might experience a “delayed onset” condition, when the medication’s effects appear slowly or unpredictably, resulting in increased “OFF” periods due to the unstable pharmacokinetics of levodopa [7]. These problems are exacerbated by dysphagia, since patients who cannot swallow well may not take medications on time or at all. In late-stage PD, dysphagia amplifies these problems: patients who cannot swallow reliably delay or omit doses, undermining adherence and symptom control [4]. Because timely dopaminergic administration is critical, such delays are linked to worse motor outcomes in clinical settings [10]. There is a clear clinical urgency for dosage forms that can circumvent swallowing difficulties and improve the reliability of drug delivery in PD.

Orally disintegrating tablets (ODTs) are a patient-centric option for dysphagia. It is solid oral formulations that rapidly disintegrate in the mouth and can be administered without water [11,12]. For PD patients with dysphagia, ODTs offer easier self-administration and potentially faster absorption onset [13]. This review first examines the impact of dysphagia in Parkinson’s disease patients and evaluates how orally disintegrating formulations of antiparkinsonian drugs address these challenges. It further explores the emerging role of 3D printing technology in introducing innovative solutions for developing easy-to-swallow Parkinson’s disease treatments. We aim to clarify the urgent need for orally disintegrating formulations in Parkinson’s care and how 3D printed formulations can overcome existing limitations.

## 2. Dysphagia in PD and Medication Challenges

Dysphagia in Parkinson’s disease on swallowing impairment is a common feature of PD, affecting an estimated 40–80% of patients at some point [14]. Early-stage dysphagia is often clinically silent; laryngeal penetration or aspiration may occur without obvious symptoms, with prevalence increasing as the disease progresses and PD severity escalates [3]. Oropharyngeal dysphagia in PD stems from bradykinesia and the incoordination of the tongue, pharynx, and esophagus swallowing muscles, resulting in slowed or incomplete bolus transit and impaired airway protection [15]. Water aspiration occurred in 48% of patients during pill intake, implying that dissolved-tablet administration could heighten aspiration risk in a flexible endoscopic study, and water aspiration was observed in 48% of PD patients during pill swallowing, suggesting that dissolving tablets in water may increase aspiration risk [16]. Dysphagia has serious consequences, including untreated dysphagia can lead to serious complications such as aspiration pneumonia, malnutrition, dehydration, and weight loss, and these complications often result in hospitalizations [17]. These happen to be the cause of about one in four PD deaths [5]. To mitigate these medication swallowing issues, alternative formulations such as orally disintegrating tablets (ODTs) have been developed, allowing patients to take medication without water [5]. Despite its lower dose, the ODT achieves comparable C_max_ and AUC values to those of the swallowed tablet because it bypasses first-pass metabolism, resulting in higher parent drug levels and significantly fewer amphetamine metabolites [18]. However, taking selegiline ODT with food reduces its bioavailability by 40%, so dosing in a fasted state is recommended [19]. By contrast, carbidopa/levodopa ODT is pharmacokinetically bioequivalent to immediate release tablets (T_max_ about 1 h). Crossover trials report no significant difference in ON time or United Parkinson’s Disease Rating Scale (UPDRS) motor scores between carbidopa/levodopa ODT and standard levodopa, indicating ODT’s main benefit is improved convenience rather than enhanced clinical effect [20]. These clinical challenges underscore the need for innovative solutions; accordingly, the next sections explore how emerging technologies from optimized ODTs to 3D printed personalized tablets aim to overcome swallowing-related limitations in PD therapy.

## 3. Patient Compliance and “Do Not Crush” Limitations

Patients diagnosed with Parkinson’s disease frequently rely on complex drug schedules. Current PD pharmacotherapy is dominated by oral formulations, notably combination carbidopa/levodopa products: IR tablets such as Sinemet^®^ and extended-release (ER) tablets or capsules. Dopamine receptor agonists, monoamine oxidase B (MAO-B) inhibitors, and dopamine receptor agonists catechol-O-methyltransferase (COMT) inhibitors [6,21,22,23], most of which are supplied as tablets or capsules.

Levodopa is time-sensitive; even minor delays or omissions may result in OFF episodes, which are associated with increased emergency visits and hospitalization as well as functional impairment [24]. Moreover, advanced Parkinson’s disease is constrained by pharmacokinetic fragility. Levodopa possesses a brief plasma half-life (approximately 90 min with carbidopa), requiring frequent administration, and as nigrostriatal degeneration progresses, the therapeutic window narrows. Even minor fluctuations in concentration that trigger end-of-dose wear-off or peak-dose dyskinesias [25,26,27].

Levodopa is absorbed in the duodenum and proximal jejunum through large neutral amino acid transporters. Thus, delayed or irregular gastric emptying significantly delays its delivery to the absorption site, leading to “delayed ON” or dose failure. Additionally, dietary protein and *Helicobacter pylori* further diminish bioavailability [28,29]. Increasing the dosage or shortening dosing intervals may maintain efficacy temporarily but elevate the risk of dyskinesia, necessitating meticulous scheduling [30]. Concurrent gastrointestinal dysmotility is prevalent in PD and further impair oral medication absorption [7,31].

Dysphagia is a major impediment to medication adherence in PD. Patients may attempt to crush tablets for easier consumption, but may pose issues with these unsupervised oral medications, which can be problematic [32]. Since crushing controlled-release tablets violate “do not crush” principles, this is directly relevant to PD pharmacotherapy. Dysphagia is associated with approximately 3–4-fold higher medication administration error rates in hospital, and PD inpatients commonly experience omitted or delayed dopaminergic doses with longer lengths of stay [33]. These circumstances foster “pill aversion” with patients delaying or avoiding doses to avert the discomfort or risk of swallowing pills.

Given these risks, clinicians increasingly employ swallow-friendly or non-oral options when available. Rotigotine transdermal patches Neupro^®^ [34] provide dopamaminergic therapy without any oral intake. ER carbidopa/levodopa capsules (Rytary/IPX066) are designed to be swallowed intact. If swallowing is difficult, the capsule may be opened and the beads sprinkled on soft food and swallowed without chewing. This preserves the intended pharmacokinetic profile, and crushing the beads undermines the release mechanism [35], which is consistent with general “do not crush” principles for modified-release products. Certain medications are also available in liquid formulations like amantadine syrup, helping with swallowing difficulties, although coverage across PD drug classes remains incomplete. In advanced cases, enteral tube delivery (Duodopa^®^) is effective but has procedure- or device-related complications [36]. Despite these workarounds, there remains a clear need for oral dosage forms that can be used without swallowing difficulty, ensuring patients receive their medications on time.

## 4. Oral Disintegrating Tablet (ODT) Availability

For Parkinson’s disease patients, orally disintegrating tablets (ODTs) can help prevent swallowing-related difficulties and reduce delays in treatment caused by traditional formulation characteristics. Currently a limited number of ODT formulations for PD are commercially available, including ODTs carbidopa/levodopa [20] and selegiline [37]).

Selegiline ODT (Zelapar^®^) dissolves on the tongue and is absorbed through the oral mucosa, bypassing first-pass metabolism [37]. This pharmacokinetic advantage lowers the risk of certain side effects and potentially improves patient compliance. Compared to a conventional swallowable tablet, selegiline ODT’s time to peak concentration (T_max_) is markedly accelerated (approximately 10–15 min compared to 40–90 min), with higher relative bioavailability. Notably, a 1.25 mg Zelapar^®^ ODT achieves plasma concentrations equivalent to 10 mg of traditional selegiline, with significantly reduced amphetamine metabolites [19,38].

Orally disintegrating tablets of carbidopa/levodopa may enhance the administration route but do not substantially modify the pharmacokinetic characteristics of levodopa. FDA bioequivalence studies show a T_max_ of approximately 1 h, like standard release tablets, with standard bioequivalence in AUC and C_max_. Randomized crossover trials also found no significant differences in ON time [39]. A multicenter study found that the carbidopa/levodopa ODT was preferred by 45% of PD patients versus 20% for the standard tablet, with easier use and less swallowing concern cited as the main reasons [20]. Given the high prevalence of dysphagia in PD (~70%), these findings support ODTs’ suitability for patients with swallowing difficulties [4].

In the United States, there has not been a commercially available ODT for carbidopa/levodopa since the approval of the Parcopa^®^ brand in 2004 but withdrawn by 2002, which is offered in dosages of 10/100, 25/100, and 25/250 mg. As of late 2023, no carbidopa/levodopa ODT remains on the US market, leaving a significant gap in care for PD patients with dysphagia. This gap matters because healthcare professionals may resort to crushing IR carbidopa/levodopa to administer with soft food, even though modified release products should not be crushed. In recent years, several “rescue” solutions that do not require swallowing have been introduced. Apomorphine sublingual film was approved by the FDA in 2020 but was voluntarily discontinued from the U.S. market in 2023. Inhaled levodopa powder was approved in 2018. Oral dispersible levodopa/benserazide (Madopar^®^) is still accessible outside of the U.S. Selegiline (Zelapar^®^) is still on the market as an ODT for PD in the U.S. However, no ODT formulation currently exists to substitute for daily levodopa tablets (Table 1). This persistent unmet need has prompted interest in novel fabrication methods, including 3D printing to create a swallow-friendly and patient-tailored PD therapeutics.

## 5. Research and Product Examples of 3D Printing ODTs

Additive manufacturing (also called “3D printing”) is being investigated as a means to produce patient-specific orally disintegrating and multi-drug tablets for PD (Table 2) [42,43]. Three-dimensional printing enables the on-demand adjustment of geometry, compartmentalization, and infill or porosity to customize dosage and drug release profiles, further permitting polypills and rapidly disintegrating “printlets” [44,45]. For PD patients, these capabilities directly address key clinical needs: a highly porous 3D printed tablet can disintegrate quickly in the mouth to provide faster onset of relief during sudden OFF episodes and is easier to swallow for those with dysphagia, while multi-drug printlets can combine medications into one unit, reducing pill burden in polypharmacy [46,47]. A regulatory pattern in neurology was established in 2015 when the FDA approved the 3D printed levetiracetam tablet (Spritam^®^) [48], a highly porous dosage form that disintegrates in approximately 11 s with a sip of liquid and is available in strengths of up to 1000 mg. Since Spritam’s approval, numerous academic and industry groups such as the specialist startup FabRx in the UK have expanded 3D pharma printing research and are actively working on this technology [49]. Table 2 summarizes research and product examples of 3D printed tablet dosage forms for Parkinson’s disease.

A recent study has begun translating these 3D printing advantages into PD therapies. Windolf et al. developed a mini-floating polypill that integrates levodopa/benserazide (LD/BZ) with pramipexole via fused-deposition modeling [50]. Mini-tablet geometries acquired gastric buoyancy and programmable release (about 75% LD/BZ at around 750 min) to prolong drug delivery and address motor fluctuations. The layer-by-layer construction of 3D printing facilitates this multipurpose design while keeping the tablets small for easier swallowing.

In a series of studies, Gültekin et al. leveraged FDM 3D printing to develop personalized pramipexole tablets with both immediate- and extended-release profiles. Their optimized IR printlets achieved rapid drug liberation (>90% released about 5 min). In contrast, a once-daily extended-release formulation provided approximately 24 h pramipexole release in vivo and attained about 107% relative bioavailability compared to a commercial ER product. Additionally, the group fabricated flexible-dose ODT, which exhibited consistent dissolution across doses (f2 value greater than 50) and remained stable over 12 months of storage, comparable to conventional tablets.

Rosch et al. demonstrated the point-of-care manufacturing of levodopa ODTs within hospital pharmacies, showing that high-quality personalized ODTs could be produced on-site without industrial-scale production [51]. The study emphasizes the necessity for drug-specific stability assessments during printing, the degradation noted for carbidopa indicates potential solutions through antioxidant excipients, lower processing temperatures, or alternate methods such as semisolid extrusion or binder jetting. Despite these challenges, the study marks an important step in tailored, 3D printed PD medications [52]. These examples illustrate how 3D printing can tailor PD medications to patient needs and potentially improve access by enabling production closer to the patient.

New concepts are arising, including microchip-integrated dosage forms that connect sensing with drug release to detect on–off states and initiate on-demand dosing, as well as implantable 3D printed hydrogels loaded with levodopa for sustained release that relieve motor symptoms. Although these techniques remain in early phases, they underscore the enormous therapeutic potential of 3D printing in Parkinson’s disease [53].

A series of studies by Gültekin et al. demonstrate the pharmaceutical technology innovations in 3D printed ODTs for Parkinson’s therapy using pramipexole as a model drug. In a 2019 study, IR low-dose pramipexole tablets were fabricated via FDM printing with a Eudragit EPO/polyethylene oxide matrix, by adjusting design parameters such as infill density, tablet thickness, and incorporating internal holes; the tablets’ disintegration and drug release were dramatically accelerated, achieving >90% release in about 5 min (versus 30 min with no holes) without the need for any added disintegrants. In a follow-up 2021 study, the same group developed extended-release 3D printed pramipexole tablets for dosing once daily, the optimal formulation (comprising 30% POLYOX™ N80, 60% Eudragit RSPO, and 10% Eudragit RL100) provided a 24 h sustained release and in vivo performance matching a commercial once-daily tablet. The 3D printed extended-release tablets displayed a 24 h plasma drug profile nearly superimposable on the marketed product (relative bioavailability: 107%) and remained stable under accelerated conditions. Most recently, Gültekin et al. applied a design-of-experiments approach to optimize a flexible dose immediate-release pramipexole printlet, confirming that polymer composition (Eudragit EPO with POLYOX N80), print thickness, and infill percentage can be tuned to modulate the release rate. Using this approach, they successfully manufactured pramipexole ODTs in both standard doses (0.25 and 1 mg) and intermediate doses (0.375, 0.5, 0.75 mg not available commercially) with consistent physico-mechanical properties and dissolution profiles. The optimized ODTs also exhibited excellent stability for 12 months at 25 °C/60% RH and 6 months at 40 °C/75% RH is comparable to that of conventional tablets. These advancements underscore that 3D printing enables patient-tailored ODTs in PD, allowing the personalization of dose and release kinetics through formulation design. Through structural engineering, the tablet maintains rapid disintegration, with drug stability on par with standard formulations.

## 6. Feasibility and Challenges of Producing 3D Printed ODTs

Integrating 3D printed ODTs into routine PD care requires overcoming practical challenges [50,54]. Considerations include regulatory and quality control frameworks and production throughput. Regulatory approval will likely hinge on system-level qualification of the entire printing process (hardware, materials, and software) under Quality-by-Design and Good Manufacturing Practice (GMP) principles, especially for point-of-care manufacturing models [55,56]. Current 3D printers have relatively low throughput and higher cost compared to mass production tablet presses, on the order of only hundreds of tablets per hour, which is a constraint for large-scale use. Three-dimensional printing’s benefits may be realized in small-batch or personalized medicine scenarios [42]. Evidence gaps in evidence are narrowing. Research on patients indicates that tablet geometry and esthetics influence swallow ability and preference [57]. At the point of care, randomized crossover data show bioequivalence for 3D printed tablets [58]. Nonetheless, alterations in formulation and process during the 3D printing procedure, along with API instability require verification [59]. Emerging pharmacokinetic data suggest that 3D printed drugs can perform as well as or even better than their marketed counterparts. For instance, a 3D printed sildenafil ODT in rats exhibited higher exposure and bioavailability than the standard product [60]. In vitro dissolution tests and pharmacokinetic modeling have shown that 3D printed sustained-release ropinirole tablets have pharmacokinetic profiles that are the same as those of commercial versions [61]. Looking forward, we anticipate that 3D printed medications aided by continued technological advances and supportive regulatory frameworks could begin entering mainstream clinical practice within the next 5–10 years [62].

## 7. Conclusions

ODTs for Parkinson’s disease patients with dysphagia improve medication adherence and safety, as evidenced by high patient preference and a reduced risk of aspiration. These formulations rapidly dissolve in the mouth and minimize swallowing challenges, enabling patients who struggle with traditional tablets to take their medication more reliably. Three-dimensional printing technologies offer capabilities to further advance this patient-centric approach. The StackDose^®^ platform, a powder 3D printing technology also facilitates novel designs, such as through size variations or an eye-catching icon on the tablet, that improve swallowability and permit the modulation of ER or IR profiles beyond the limits of conventional tablets [63]. A recent animal study using StackDose^®^ revealed that a 3D printed sildenafil ODT achieved nearly 100% dissolution within 5 min at both pH 1.2 and 4.5 and demonstrated significantly enhanced systemic bioavailability compared to the reference tablet increases of 274.8% with omeprazole pretreatment and 144% without, highlighting its performance advantage under variable gastric conditions [60]. In the short term, 3D printed ODT solutions are only beneficial in a few select cases, such as personal treatment for those with severe dysphagia due to current technology and regulatory limits. In the long term, as 3D printing techniques improve, 3D printed ODTs are expected to enable safer, more individualized drug delivery in PD, enhancing patients’ compliance and reducing caregiver burdens. Achieving this vision demands strong interdisciplinary collaboration across pharmacy, medicine, and engineering, alongside strong safety and regulatory frameworks with clear guidance and process standardization and solutions to drug stability challenges in printing. With continued innovation and rigorous validation, 3D printed levodopa ODTs could enter clinical use within decades.

## Figures and Tables

**Table 1:** Current and emerging formulations for Parkinson’s disease and their features.

Medication/Category	Conventional Form	Swallow-Friendly Formulations	Notes (Regulatory Status)
Carbidopa/Levodopa (CD/LD)	IR tablets ER tablets/capsules Intestinal gel infusion Inhalation powder	IR ODT: Parcopa^®^, discontinued in US. Inhaled: levodopa powder Inbrija^®^, FDA-approved in 2018.	Parcopa^®^ ODT-approved in 2004 [40], removed 2022. No current CD/LD ODT in US. Inhaled levodopa bypasses gut, used for OFF periods.
Selegiline (MAO-B inhibitor)	Tablets Capsule Transdermal patch	ODT: Zelapar^®^ (1.25 mg), FDA-approved in 2006 [19].	Zelapar^®^ ODT dissolves in seconds, increasing bioavailability and faster onset compared to 5 mg tablet. Used as adjunct in PD.
Apomorphine (Dopamine agonist)	Subcutaneous injection (Apokyn^®^) Pump infusion	Sublingual film: Kynmobi™ dissolves under tongue, FDA-approved in 2020 [41].	Kynmobi™ film provides on-demand rescue for OFF episodes without needles. Not the ODT dosage form, but the concept is like ODT: no swallowing required.
Others (pramipexole, ropinirole, entacapone,)	Oral tablets (IR/ER formulations)	No ODT forms are available for these as of 2025. Some are available as liquid formulations via compounding or abroad.	ER formulations must not be crushed [15]. Crushing can cause dose dumping. Patients with dysphagia may consider switching to IR or liquid forms.

IR, immediate release; ER, extended release.

**Table 2:** Recent research on the application of 3D printed ODTs for Parkinson’s disease medications.

Research	Manufacturing Procedure	Formulations/Dosage Forms	Key Findings
Windolf et al. [50].	FDM printing assisted by hot melt extrusion (multi-material and multi-layer)	3D printed mini-floating polypill: prototype combining levodopa/benserazide, pramipexole in one small pill	Research stage shows feasibility of personalized dosing and dysphagia friendly design. Rapid pramipexole for early-morning OFF. ER levodopa/benserazide > 12 h, buoyant design prolongs gastric residence, and levodopa 15~180 mg with similar release enables personalized dosing.
Rosch et al. [51]	Direct powder extrusion, DPE	carbidopa/levodopa ODT	Carbidopa degrades during direct powder printing at 180 °C in a hospital setting, highlighting API-specific process compatibility.

FDM, fused deposition modeling; SSE, semisolid extrusion; API, active pharmaceutical ingredient.

## Data Availability

No new data were created or analyzed in this study. Data sharing is not applicable to this article.

## Abbreviations

The following abbreviations are used in this manuscript:

PD	Parkinson’s disease;
ODT	Orally disintegrating tablet;
CD/LD	Carbidopa/Levodopa;
IR	Immediate release;
ER	Extended release;
API	Active pharmaceutical ingredient;
LD/BZ	Levodopa/benserazide;
QbD	Quality by design;
GMP	Good manufacturing products.
